# Melanoma in the Italian Population and Regional Environmental Influences: A National Retrospective Survey on 2001–2008 Hospitalization Records

**DOI:** 10.3390/ijerph120809102

**Published:** 2015-08-05

**Authors:** Prisco Piscitelli, Cosimo Neglia, Andrea Falco, Matteo Rivezzi, Nadia Agnello, Alberto Argentiero, Giovanna Chitano, Chiara Distante, Giulia Della Rosa, Giorgia Vinci, Antonella De Donno, Alessandro Distante, Antonella Romanini

**Affiliations:** 1 Southern Italy Hospital Institute, IOS/Coleman Ltd., Naples 80100, Italy; E-Mails: falco.and@gmail.com (A.F.); matteo.rivezzi@gmail.com (M.R.); 2 Euro Mediterranean Biomedical Scientific Institute, Brindisi 72100, Italy; E-Mails: neglia@isbem.it (C.N.); agnello@isbem.it (N.A.); albertoargentiero@isbem.it (A.A.); chitano@isbem.it (G.C.); chiara.distante@gmail.com (C.D.); dellarosa.giulia@gmail.com (G.D.R.); distante@isbem.it (A.D.); 3 Department of Medical Sciences, Federico II University, Naples 80132, Italy; E-Mail: giorgia.vinci@hotmail.it; 4 Department of Science, Biotechnology and Environment (DISTEBA), University of Salento, Lecce 73100, Italy; E-Mail: antonella.dedonno@unisalento.it; 5 Melanoma Unit, Pisa University Hospital, Pisa 56126, Italy; E-Mail: a.romanini@ao-pisa.toscana.it

**Keywords:** Melanoma, incidence, hospitalizations, regions, environment, environmental factors

## Abstract

*Objective*: To assess the burden of regional environmental factors influencing the incidence of Melanoma in the Italian population and overcome the problem of partial population coverage by local cancer registries and thematic archives. *Methods*: We analyzed the Italian national hospitalization records from 2001 to 2008 provided by the Ministry of Health, excluding hospital re-admissions of the same patients, in order to assess the occurrence of Melanoma over a 8-year period. Data were presented by age groups (absolute number of cases from 20 to ≥80 years old) and per Region (rates per 100,000 inhabitants) for each year. *Results*: The overall number of new hospitalizations due to malignant Melanoma increased by 16.8% from 2001 (n = 4846) to 2008 (n = 5823), with the rate per 100,000 inhabitants passing from 10.5 to almost 12.0 at a national level. The majority of new diagnoses of malignant Melanoma was observed in two age groups: 61–70 years old (from 979 in 2001 up to 1209 in 2008, corresponding to 15.1 and 18.1 new cases per 100,000 inhabitants, respectively) and 71–80 years old (from 954 in 2001 up to 1141 in 2008, corresponding to 19.5 and 21.8 new cases per 100,000 inhabitants, respectively). The number of hospitalizations due to Melanoma increased in all age groups with the only exception of the youngest patients aged 20–30 years old. The highest increases over the 8-year period were observed in people aged ≥81 years old (+34%), 61–70 years old (+20%) and surprisingly in the age group 31–40 years old (+17%). Southern Regions showed lower hospitalization rates compared to Northern Italy and Region Lazio. The highest increases between 2001 and 2008 were observed in Trentino/Alto Adige, Friuli Venezia Giulia, Valla d’Aosta and Veneto Region. *Conclusions*: Hospitalizations due to malignant Melanoma in Italy seem to be influenced by environmental or population-related factors showing a decreasing incidence rate from the Northern to Southern Regions.

## 1. Introduction

Cutaneous malignant Melanoma (CMM) is a potentially lethal form of skin cancer. Although it accounts for only 3% to 5% of all skin cancers, it is responsible for approximately 75 percent of all skin cancer deaths [1]. CMM results from the malignant transformation of melanocytes, which are the pigment-producing cells responsible for the color of skin. The key triggers leading to malignant transformation of melanocytes have yet to be fully elucidated, but are known to be multifactorial and include UV radiation damage and genetic susceptibility. Melanoma was diagnosed in approximately 85,000 people globally in 2008 [2] and is in general confined to economically developed countries. In particular, there is a high incidence in countries with fair-skinned populations, such as Northern Europe, the US, Australia, New Zealand, and South Africa. The incidence of CMM is highest in white persons. This population is approximately 20 times more likely to develop the disease than black persons. It is rare in young persons, with only 2 % of Melanomas occurring in persons younger than 20 years and approximately 0.3 percent in children younger than 14 years [3]. Persons with an increased number of moles, dysplastic (also called atypical) nevi, or a family history of this disease are at increased risk compared with the general population.

According to the Italian Cancer Society, there is an annual average incidence of 12.5 new skin Melanoma diagnoses per 100,000 males and 13.1 per 100,000 females [4]. Incidence rates for skin Melanoma vary sensibly across Italy, with a decreasing trend moving from North to the South. Worldwide, Melanoma incidence and death rate have progressively increased during the last 30 years [5].

An important tool to assist in the evaluation of potential Melanomas for patients and health care professionals is the ABCDE mnemonic, which takes into account asymmetry, border irregularities, color variation, diameter, and evolution. Any suspicious pigmented lesion should be biopsied to determine the histologic depth of lesion penetration, which is known as the Breslow depth. The Breslow depth is the most important prognostic parameter in evaluating the primary tumor. Early detection and treatment can lead to longer survival. According to the American Academy of Dermatology Association and the Society for Investigative Dermatology, the estimated total direct cost associated with the treatment of Melanoma in 2004 was $291million, of which $101 million were for office visits, $76 million for hospital outpatient treatment, $78 million for prescription drugs, $35 million for hospital inpatient treatment and $1 million for emergency room treatment [6].

In Italy, a network of population-based local cancer registries has been established (Italian Association of Cancer Registries, AIRTUM, Roma, Italy) in order to set high qualitative standards in data collection that can result in reliable reports. However, the AIRTUM CRs cover about 35% of the Italian population, (19 million people out of 61 million inhabitants), with a remarkable difference in CRs population coverage among Northern (50.2%), Central (25.5%) and Southern areas of the Country (17.9%) [7].

Also for Melanoma, as well as for mesothelioma, advanced experiences of thematic registries are also available, despite being limited to some hospitals or regions. Because no complete data are available about the occurrence of Melanoma in the entire Italian population, our aim was to provide some first data about hospitalizations due to main diagnosis of Melanoma in Italy. Although Cancer Registries remain the gold standard methodology to collect epidemiological information on cancer at local level, we attempted to estimate the overall burden of Melanoma at regional level and for age groups by analyzing hospitalization records, which have already been used for this purpose in our previous studies about breast cancer and in other researches [8,9,10,11].

## 2. Experimental Section

### Methods 

Information concerning all hospitalizations occurring in Italian public and private care setting are registered in hospital discharge records (HDR), which are collected in the Italian Ministry of Health national hospitalization database (SDO). These information are anonymous and include: region and hospital where the patients have been hospitalized, type of hospitalization (ordinary admission or day hospital), region and province where the patient come from, local health authority (ASL) who is paying for the hospitalization costs, patient’s age, gender, main diagnosis, secondary diagnoses (up to five), procedures performed (main procedure and up to five additional procedures), diagnosis related group (DRG) and length of the hospitalization. It should be noticed that in the national hospitalization database, those people admitted at hospitals located in a region or province different from those where the patients live, are classified according to their home address. Hospitalization records are kept at a central level by the Ministry of Health from 1999 to date, but some records concerning the years 1999 and 2000 are missing and the national hospitalization database has been fully implemented for all Italian regions only from 2001. The Italian Ministry of Health has officially provided the full database concerning all hospitalizations occurred in Italy between 2001 and 2008 due to several diagnosis of Melanoma. The quality of these data is known to be very high and certified at central level by the Ministry of Health, with completeness and reliability of records (in terms of correspondence between hospitalizations and individual social security numbers, but also in terms of absence of errors or missing data) varying from 95.6% (year 2001) and 99.8% (for year 2008), respectively, as reported in our previous studies [10,11].

Our dataset included all hospitalized patients with major diagnosis of skin Melanoma based on the the International Classification of Diseases, Ninth Revision, Clinical Modification (ICD-9-CM) code 172 (all extensions). Patients with Melanoma in other parts of the body were not captured in the analysis.

Based on social security numbers (which were treated anonymously), the Ministry of Health has allowed us to exclude all hospital re-admissions of the same patient over the entire study period, in order to minimize possible bias related to the overlapping between prevalent and incident cancer cases. To exclude hospital re-admissions from our analysis, we have considered as “index” hospitalization the first hospital admission of the patients for which repeated hospitalizations were recorded over the entire study period (2001-2008). Patients presenting the same social security number (treated anonymously) and the same major diagnosis were considered as the same person, and they were computed only one time. This methodology has been already used and certified by the Environment Protection Agency of Piemonte Region for the assessment of population heath indicators [12].

The absolute frequencies (number of hospitalizations) were computed for each Italian Region (Reg) and Province (Prov), by sex (S), year (y), and 10-year age groups (x):

$$n_{y,x}^{S}\left(Reg\;or\;Prov\right)$$

The standardized hospitalization rate per 100,000 inhabitants was computed by referring to the Italian population $P_{y,x}^{S}\left(IT\right)$ of year 2001 (y) per age group (x) and sex (S):

HRSy,x=∑xhRSy,x*Py,xS(IT)∑xPy,xS(IT) × 100

Data were analyzed and processed using the Stata (StataCorp, College Station, TX, USA) and Excel (Microsoft, Redmond, WA, USA) softwares. Age and sex standardized rates per Region and Province were calculated based on population data provided by the Italian National Institute for Statistics (ISTAT) for the year 2001. The results of the analyses in this first paper have been studied as cumulative data (all tumours) per each Italian Region and Province according to sex and age groups from 20 to 100 years old. Data are specifically presented per Region as absolute number of hospitalizations and standardized hospitalization rate for the each year from 2001 to 2008.

## 3. Results and Discussion

**Figure 1:**
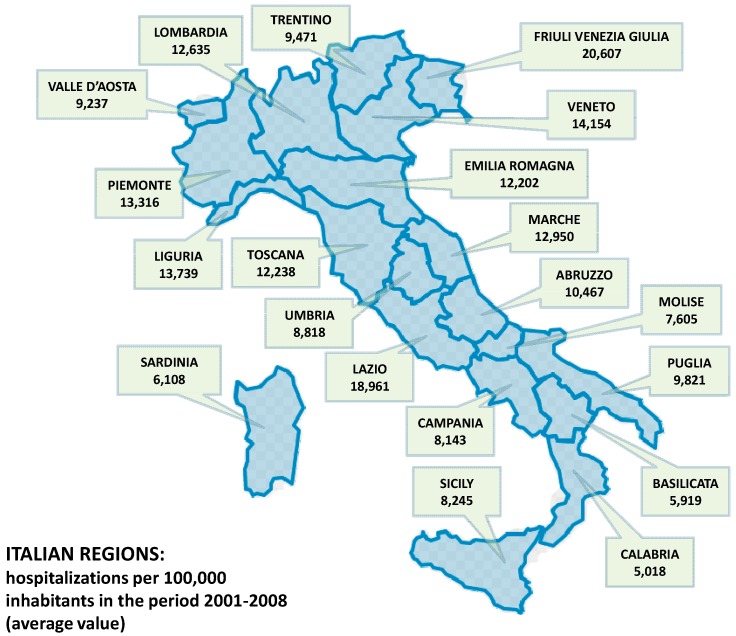
Standardized Hospitalization rate per 100,000 inhabitants (malignant skin Melanoma) displayed for each Italian region as average value 2001–2008.

**Figure 2:**
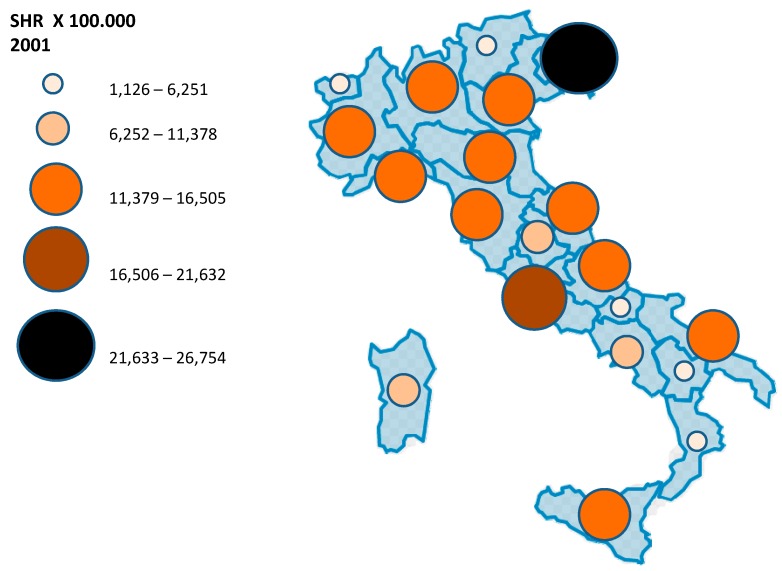

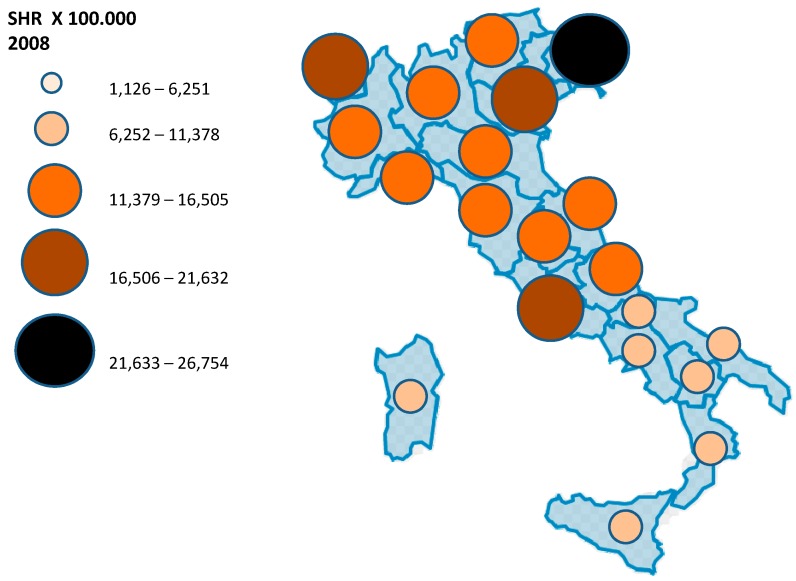
Differences in the hospitalizations due to malignant skin Melanoma between 2001 and 2008 in the Italian Regions.

As Reported in Table 1, the overall number of new hospitalizations due to main diagnosis of malignant Melanoma increased by 16.8% from 2001 (n = 4846) to 2008 (n = 5823), with new hospitalizations per 100,000 inhabitants passing from 10.5 to almost 12.0 at the national level (Table 2). The majority of new diagnoses of malignant Melanoma was observed in two age groups: 61–70 years old (from 979 in 2001 up to 1209 in 2008, corresponding to 15.1 and 18.1 new cases per 100,000 inhabitants, respectively) and 71–80 years old (from 954 in 2001 up to 1141 in 2008, corresponding to 19.5 and 21.8 new cases per 100,000 inhabitants, respectively). The number of hospitalization due to malignant Melanoma increased in all age groups with the only positive exception of the youngest patients aged 20–30 years old. The highest increases over the 8-year period were observed in people aged ≥81 years old (+34%), 61–70 years old (+20%) and surprisingly in the age group 31–40 years old (+17%). Table 3 summarizes the number of hospitalizations per 100,000 inhabitants in the Italian regions and the corresponding standardized hospitalization rates, showing that the Southern regions and islands (Sicily and Sardinia) have lower rates compared to northern regions (8.4 and 13.3, respectively in year 2008). Southern regions presented also the lowest increase over the 8-year period. The number of hospitalizations recorded in the Lazio region were higher than those observed in other Central Italian regions. Friuli Venezia Giulia, Lazio, Veneto, and Valle d’Aosta presented the highest hospitalization rates due to main diagnosis of Melanoma (ranging from 16 to 24 per 100,000 in 2008), while Apulia was the region where the highest number of cases were observed in Southern Italy (Table 3). Figure 1 summarizes the average (2001–2008) hospitalization rates per 100,000 inhabitants for each region. The highest increases in the number of hospitalizations between 2001 and 2008 were observed in the Trentino/Alto Adige, Friuli Venezia Giulia, Valla d’Aosta and Veneto regions, as shown in Table 3 and displayed in Figure 2. Table 4 shows the Standardized Hospitalizations Rate (SHR) per 100,000 inhabitants in Italian regions per year (2001–2008) and age groups, highlighting that people aged 71–80 and >80 are usually more affected by skin Melanoma. However, the incidence in younger people is different according to the regions where people live. 

**Table 1:** Number of new hospitalizations due to main diagnosis of Malignant Skin Melanoma in Italy for age groups and years (2001–2008). Data provided by the Italian ministry of health.

Age Group	2001	2002	2003	2004	2005	2006	2007	2008
20 to 30 years old	334	278	242	329	271	297	301	286
31 to 40 years old	600	614	549	623	659	673	728	729
41 to 50 years old	714	644	598	681	748	843	844	898
51 to 60 years old	872	817	783	857	858	930	942	964
61 to 70 years old	979	963	968	940	1.007	1.074	1.197	1.209
71 to 80 years old	954	847	865	844	895	1.030	1.024	1.141
≥81 years old	393	418	416	433	473	477	581	596
**TOTAL**	**4.846**	**4.581**	**4.421**	**4.707**	**4.911**	**5.324**	**5.617**	**5.823**

**Table 2:** Hospitalizations per 100,000 inhabitants due to main diagnosis of Malignant Skin Melanoma in Italy for age groups and years (2001–2008). Data provided by the Italian ministry of health.

Age Group	2001	2002	2003	2004	2005	2006	2007	2008
20 to 30 years old	3.92	3.35	2.96	4.08	3.46	3.89	4.00	3.83
31 to 40 years old	6.56	6.63	5.83	6.53	6.93	7.10	7.71	7.78
41 to 50 years old	9.39	8.18	7.38	8.15	8.69	9.54	9.29	9.66
51 to 60 years old	12.26	11.42	10.81	11.68	11.51	12.20	12.36	12.61
61 to 70 years old	15.16	14.87	14.91	14.48	15.59	16.72	18.22	18.09
71 to 80 years old	19.56	17.09	17.31	16.75	17.62	20.01	19.76	21.80
≥81 years old	18.33	18.55	17.58	17.34	15.72	17.49	20.47	20.23
**TOTAL**	**10.56**	**9.90**	**9.44**	**9.95**	**10.24**	**11.12**	**11.64**	**11.97**

**Table 3:** Absolute number (N) and Standardized Hospitalizations Rate (SHR) per 100.000 inhabitants due to main diagnosis of Malignant Skin Melanoma in Italian Regions per year (2001–2008).

Region	2001		2002		2003		2004		2005		2006		2007		2008	
	N	SHR	N	SHR	N	SHR	N	SHR	N	SHR	N	SHR	N	SHR	N	SHR
Piemonte	385	12,164	419	13,172	385	11,982	415	12,767	471	14,441	432	13,271	457	13,914	489	14,819
Val d'Aosta	−	NA *	−	NA *	12	13,235	8	8,772	7	7,588	11	11,909	8	8,598	17	18,220
Lombardia	814	12,202	832	12,359	853	12,482	808	11,668	876	12,542	953	13,612	983	13,912	876	12,303
Trentino	40	6,041	55	8,199	45	6,621	71	10,313	78	11,179	59	8,406	87	12,217	92	12,790
Veneto	432	12,971	464	13,782	418	12,246	434	12,593	408	11,719	536	15,374	582	16,487	643	18,056
Friuli	190	21,140	162	17,904	126	13,860	184	20,184	164	17,887	196	21,464	236	25,667	247	26,754
Liguria	185	15,290	190	15,685	146	12,022	158	12,919	145	11,720	155	12,623	177	14,418	187	15,230
Emilia	376	12,425	330	10,786	315	10,195	397	12,686	387	12,261	406	12,837	394	12,335	455	14,092
Toscana	388	14,686	334	12,544	260	9,642	270	9,948	281	10,305	359	13,150	392	14,231	371	13,395
Umbria	53	8,612	54	8,650	42	6,630	43	6,703	62	9,560	69	10,599	55	8,346	76	11,445
Marche	128	11,781	139	12,624	134	12,023	135	12,007	126	11,142	171	15,085	166	14,486	167	14,452
Lazio	648	17,422	639	17,040	705	18,584	700	18,228	799	20,637	799	19,956	800	19,752	822	20,073
Abruzzo	102	11,182	89	9,620	82	8,757	104	10,982	107	11,207	109	11,375	87	8,969	114	11,644
Molise	11	4,790	20	8,637	15	6,430	17	7,281	20	8,554	20	8,567	22	9,370	17	7,209
Campania	281	7,555	244	6,507	267	7,038	301	7,838	308	7,955	325	8,117	402	9,925	418	10,207
Puglia	322	11,644	248	8,899	227	8,071	252	8,879	265	9,302	264	9,254	322	11,235	325	11,286
Basilicata	24	5,743	19	4,529	15	3,557	30	7,089	26	6,135	27	6,393	22	5,193	37	8,710
Calabria	80	5,813	58	4,187	56	4,010	62	4,428	66	4,695	82	5,838	66	4,656	93	6,517
Sicily	386	11,367	281	8,225	227	6,570	264	7,624	249	7,150	274	7,853	314	8,940	290	8,229
Sardinia	−	NA *	−	NA *	91	7,636	54	4,496	66	5,434	77	6,310	45	3,659	87	7,034

* NA: Not Available because Sardinia and Valle d’Aosta did not provide hospitalization records for 2001 and 2002.

**Table 4:** Standardized Hospitalizations Rate (SHR) per 100,000 inhabitants due to main diagnosis of Malignant Skin Melanoma in Italian Regions per year (2001–2008) and age groups.

Age Group	Region	2001	2002	2003	2004	2005	2006	2007	2008
*20–30*	Piemonte	4,25	3,78	3,41	4,21	3,61	5,00	5,25	3,27
*31–40*	Piemonte	6,30	9,83	7,41	8,11	9,27	7,27	7,45	7,72
*41–50*	Piemonte	9,89	11,59	10,92	10,78	10,45	9,21	10,92	10,03
*51–60*	Piemonte	12,45	14,33	15,31	12,82	14,10	13,28	12,96	14,52
*61–70*	Piemonte	20,15	16,48	15,50	17,51	22,69	21,37	23,02	24,18
*71–80*	Piemonte	20,09	23,94	19,51	25,84	29,52	25,60	28,17	28,83
*>80*	Piemonte	11,01	42,32	34,64	40,43	49,46	41,85	41,23	47,76
*20–30*	Val d’Aosta	NA *	NA *	7,77	0,00	0,00	0,00	0,00	17,44
*31–40*	Val d’Aosta	NA *	0,00	0,00	5,25	5,28	5,36	10,83	5,55
*41–* *50*	Val d’Aosta	NA *	NA *	6,22	6,04	17,35	5,62	10,87	10,60
*51–60*	Val d’Aosta	NA *	0,00	34,70	0,00	0,00	13,24	13,21	33,08
*61–70*	Val d’Aosta	NA *	0,00	15,35	15,28	7,62	38,35	7,58	37,61
*71–80*	Val d’Aosta	NA *	NA *	30,34	39,94	0,00	39,42	19,71	39,11
*>80*	Val d’Aosta	NA *	NA *	19,42	73,80	17,84	17,31	16,55	31,78
*20–30*	Lombardia	3,78	3,75	3,34	5,11	3,62	4,38	5,44	3,41
*31–40*	Lombardia	7,08	8,31	7,72	7,75	6,87	7,94	8,54	7,31
*41–50*	Lombardia	11,21	9,73	9,29	8,87	10,54	10,72	10,21	10,19
*51–60*	Lombardia	12,50	13,63	11,62	13,03	14,83	16,06	15,65	13,16
*61–70*	Lombardia	16,91	18,12	18,77	17,37	18,90	21,39	21,39	19,38
*71–80*	Lombardia	25,12	26,53	28,36	22,90	28,11	26,46	27,45	26,78
*>80*	Lombardia	23,89	47,63	58,20	39,77	43,95	44,64	41,81	39,00
*20–30*	Trentino	0,00	1,85	0,94	8,59	6,79	3,91	3,91	3,91
*31–40*	Trentino	2,75	6,06	4,03	5,38	4,75	4,81	6,25	4,24
*41–50*	Trentino	3,44	7,47	4,80	3,84	12,53	4,97	6,87	12,02
*51–60*	Trentino	4,86	8,75	9,65	10,47	11,11	12,98	18,30	17,10
*61–70*	Trentino	15,22	10,26	11,11	19,57	19,31	18,27	15,59	18,42
*71–80*	Trentino	17,50	21,85	13,12	21,89	27,61	17,37	23,06	25,65
*>80*	Trentino	6,16	34,75	21,68	27,99	31,40	11,53	39,87	38,42
*20–30*	Veneto	6,20	3,78	6,38	5,74	5,11	6,32	6,59	5,80
*31–40*	Veneto	8,07	9,88	8,48	8,33	8,76	11,20	11,25	13,91
*41–50*	Veneto	11,74	10,69	8,65	9,77	9,56	13,66	14,76	15,44
*51–60*	Veneto	16,28	19,54	15,65	15,14	13,85	16,34	17,70	19,91
*61–70*	Veneto	17,00	23,60	18,82	16,96	15,83	25,00	24,68	27,05
*71–80*	Veneto	24,62	20,42	26,57	20,55	17,60	29,00	31,62	30,36
*>80*	Veneto	14,22	32,92	37,61	34,09	29,27	39,32	40,83	42,22
*20–30*	Friuli V. G.	8,25	8,67	3,28	17,16	4,43	8,25	11,22	10,47
*31–40*	Friuli V. G.	13,85	11,35	10,10	12,95	11,93	12,09	16,30	20,73
*41–50*	Friuli V. G.	18,64	14,14	11,15	22,25	18,45	19,73	16,82	19,75
*51–60*	Friuli V. G.	27,28	24,66	13,35	20,04	19,06	24,22	27,84	29,42
*61–70*	Friuli V. G.	29,99	24,15	20,87	24,91	26,83	29,24	40,32	36,50
*71–80*	Friuli V. G.	34,11	28,60	23,17	24,30	37,09	36,02	60,00	46,50
*>80*	Friuli V. G.	18,95	51,86	35,82	34,30	41,91	53,13	62,28	57,04
*20–30*	Liguria	3,93	4,86	4,36	5,26	4,55	4,72	5,64	3,24
*31–40*	Liguria	9,29	8,79	6,47	5,97	5,50	8,45	8,16	5,91
*41–50*	Liguria	12,32	13,07	9,13	6,86	7,93	8,62	7,08	9,12
*51–60*	Liguria	16,97	16,60	10,89	16,53	12,62	12,89	10,46	15,25
*61–70*	Liguria	17,87	22,31	19,40	15,02	19,67	18,53	26,52	18,92
*71–80*	Liguria	29,10	28,82	24,02	23,33	21,05	20,50	24,65	26,34
*>80*	Liguria	19,85	40,87	35,44	36,82	26,50	33,15	27,80	48,11
*20–30*	Emilia R.	5,36	3,97	3,61	6,08	4,74	4,89	5,47	4,17
*31–40*	Emilia R.	7,98	6,14	5,19	8,13	7,28	9,50	6,63	6,79
*41–50*	Emilia R.	9,75	9,42	7,94	10,20	10,74	10,09	11,59	9,63
*51–60*	Emilia R.	14,20	11,35	11,66	13,60	12,51	11,62	10,38	13,56
*61-70*	Emilia R.	16,78	15,34	14,94	15,27	17,00	18,14	16,48	23,19
*71–80*	Emilia R.	19,82	17,07	15,54	22,25	20,35	21,50	20,22	31,13
*>80*	Emilia R.	20,07	30,72	24,89	37,51	31,68	32,06	33,53	41,01
*20–30*	Toscana	4,07	5,06	4,89	4,45	5,17	5,05	6,01	5,47
*31–40*	Toscana	6,46	8,95	5,05	6,93	8,08	6,77	9,66	7,80
*41–50*	Toscana	12,97	8,74	6,88	8,19	8,12	11,33	10,57	9,17
*51–60*	Toscana	19,27	15,19	9,47	9,87	10,20	13,81	16,68	13,98
*61–70*	Toscana	18,30	15,20	15,16	14,91	12,80	18,86	19,94	17,31
*71–80*	Toscana	24,94	18,23	20,69	18,12	15,49	23,25	26,61	25,20
*>80*	Toscana	27,25	36,16	30,86	26,17	28,07	33,42	33,33	36,31
*20–30*	Umbria	0,00	2,17	0,00	1,11	3,36	2,29	0,00	5,72
*31–40*	Umbria	6,26	6,14	5,98	3,37	6,68	3,35	4,96	7,45
*41–50*	Umbria	6,06	6,87	5,69	5,53	2,67	12,99	7,55	12,26
*51–60*	Umbria	8,29	11,25	7,09	7,96	11,68	9,73	10,66	14,59
*61–70*	Umbria	18,20	10,68	5,35	4,28	14,01	14,10	11,82	13,92
*71–80*	Umbria	12,32	15,88	7,35	13,50	20,98	17,30	14,93	18,57
*>80*	Umbria	12,61	30,17	24,07	26,77	29,73	30,29	23,69	17,69
*20–30*	Marche	3,48	3,55	3,58	7,90	1,87	5,13	4,52	3,91
*31–40*	Marche	8,30	6,71	5,64	5,63	6,55	8,43	6,06	8,38
*41–50*	Marche	11,73	9,25	5,27	9,73	6,46	12,19	11,37	8,32
*51–60*	Marche	13,50	10,83	10,04	12,72	12,50	13,05	14,59	15,59
*61–70*	Marche	12,95	19,75	19,92	20,04	18,43	21,16	19,08	24,19
*71–80*	Marche	22,78	21,09	21,91	27,72	17,51	29,03	21,09	28,83
*>80*	Marche	14,51	48,75	45,14	34,28	31,68	46,06	41,22	34,54
*20–30*	Lazio	9,46	8,68	8,42	10,93	9,68	9,49	8,20	9,66
*31–40*	Lazio	9,74	10,31	13,22	15,66	17,12	16,89	16,64	16,16
*41–50*	Lazio	14,29	16,61	13,90	15,73	19,58	17,58	16,57	17,11
*51–60*	Lazio	20,02	20,07	20,68	22,91	22,04	20,93	23,72	22,01
*61–70*	Lazio	28,36	24,79	30,41	24,47	27,61	26,00	27,70	27,83
*71–80*	Lazio	27,79	29,62	34,74	26,97	30,70	37,40	31,81	37,67
*>80*	Lazio	26,16	52,49	59,15	41,53	53,27	62,49	45,63	56,36
*20–30*	Abruzzo	4,57	5,31	0,00	4,71	4,12	4,20	4,20	5,64
*31–40*	Abruzzo	6,26	4,49	4,42	6,03	8,26	3,88	7,74	9,45
*41–50*	Abruzzo	7,84	6,37	9,28	10,25	8,21	10,86	5,55	7,04
*51–60*	Abruzzo	12,54	9,35	7,02	13,71	9,28	8,56	5,21	9,06
*61–70*	Abruzzo	12,98	15,31	11,68	11,76	15,86	11,27	14,30	14,04
*71–80*	Abruzzo	19,00	18,76	23,19	19,43	10,58	29,81	17,53	19,18
*>80*	Abruzzo	31,93	41,69	41,36	39,22	27,11	50,20	19,30	42,58
*20–30*	Molise	0,00	5,17	2,61	8,01	2,74	2,80	5,65	0,00
*31–40*	Molise	9,36	13,97	6,95	2,34	4,71	4,77	9,58	4,85
*41–50*	Molise	10,29	5,06	4,95	9,79	12,02	7,12	4,66	11,49
*51–60*	Molise	3,06	2,96	2,86	2,78	8,06	5,31	15,80	5,26
*61–70*	Molise	5,97	12,16	12,62	13,03	3,35	6,88	10,28	3,36
*71–80*	Molise	0,00	13,83	13,78	17,03	10,21	13,60	13,64	6,83
*>80*	Molise	0,00	33,56	25,50	12,24	41,44	34,29	21,93	21,02
*20–30*	Campania	4,17	3,00	3,03	3,24	4,75	6,86	4,46	4,47
*31–40*	Campania	6,10	5,67	3,81	4,42	5,71	4,10	6,83	6,84
*41–50*	Campania	7,30	4,98	6,15	8,22	6,71	7,24	6,90	8,68
*51–60*	Campania	8,99	8,10	10,01	8,84	10,22	9,15	11,78	11,01
*61–70*	Campania	8,02	8,14	10,38	12,61	10,07	9,27	14,29	14,97
*71–80*	Campania	12,61	11,29	14,50	13,61	11,69	15,10	20,83	18,50
*>80*	Campania	9,51	21,00	20,94	22,74	19,80	27,34	32,59	27,60
*20–30*	Puglia	3,24	3,66	3,33	3,97	3,13	2,83	5,20	6,61
*31–40*	Puglia	5,03	6,61	4,62	5,15	4,63	5,55	5,74	7,23
*41–50*	Puglia	13,59	7,91	8,56	4,78	8,15	9,71	10,06	9,51
*51–60*	Puglia	15,37	8,99	9,76	11,98	10,49	13,99	11,57	10,74
*61–70*	Puglia	17,66	18,49	11,55	15,10	17,59	14,69	18,56	14,78
*71–80*	Puglia	23,90	17,28	15,99	19,93	19,97	16,32	24,45	21,80
*>80*	Puglia	14,37	26,10	27,56	35,05	31,01	24,46	36,58	39,98
*20–30*	Basilicata	0,00	0,00	1,34	2,73	2,80	1,44	4,41	3,00
*31–40*	Basilicata	3,62	0,00	0,00	1,23	3,74	3,78	3,82	3,88
*41–50*	Basilicata	2,79	2,73	1,33	5,25	3,86	6,37	5,02	8,66
*51–60*	Basilicata	12,08	5,03	4,88	4,72	6,05	8,95	4,41	10,27
*61–70*	Basilicata	9,99	13,64	7,07	18,30	9,46	19,48	9,82	13,61
*71–80*	Basilicata	10,50	18,67	4,12	16,11	9,95	15,83	7,93	13,77
*>80*	Basilicata	4,90	18,39	4,34	32,68	19,86	7,56	7,19	10,23
*20–30*	Calabria	2,23	1,89	0,76	0,77	1,19	1,62	2,04	4,11
*31–40*	Calabria	3,31	2,21	2,93	3,70	6,36	3,41	4,16	5,70
*41–50*	Calabria	4,17	2,86	2,80	3,56	3,11	3,08	4,55	5,23
*51–60*	Calabria	7,00	2,93	3,35	6,05	1,80	8,46	3,98	8,34
*61–70*	Calabria	5,39	5,48	5,59	3,98	4,62	6,44	5,19	5,06
*71–80*	Calabria	13,10	11,49	6,67	7,89	7,17	7,74	9,00	9,59
*>80*	Calabria	18,48	30,50	20,82	17,01	21,13	26,10	12,49	17,52
*20–30*	Sicilia	9,28	4,88	2,53	3,38	3,45	3,02	2,53	2,55
*31–40*	Sicilia	11,57	4,88	3,52	4,57	4,14	5,37	7,48	6,34
*41–50*	Sicilia	9,02	6,35	4,40	5,78	5,37	6,86	5,68	7,12
*51–60*	Sicilia	10,38	6,96	8,12	9,45	7,42	8,43	7,72	8,42
*61–70*	Sicilia	14,19	9,67	10,67	10,82	12,47	10,20	17,79	13,54
*71–80*	Sicilia	16,28	15,76	14,27	13,93	13,54	16,93	21,63	15,62
*>80*	Sicilia	11,51	37,89	26,12	27,56	25,64	28,91	27,65	21,31
*20–30*	Sardegna	NA *	NA *	1,40	0,48	2,00	2,07	0,53	2,19
*31–40*	Sardegna	NA *	NA *	4,53	2,87	5,74	4,11	2,89	4,16
*41–50*	Sardegna	NA *	NA *	5,00	4,46	2,61	5,97	4,61	4,54
*51–60*	Sardegna	NA *	NA *	12,15	7,24	7,04	8,41	2,93	5,80
*61–70*	Sardegna	NA *	NA *	16,02	5,70	7,44	6,72	4,18	14,03
*71–80*	Sardegna	NA *	NA *	15,92	7,80	10,15	6,70	4,11	17,09
*>80*	Sardegna	NA *	NA *	23,22	13,51	17,68	15,48	13,31	21,28

* NA: Not available because Sardinia and Valle d’Aosta did not provided hospitalization records for years 2001 and 2002.

### Discussion

Melanoma is the deadliest form of skin cancer. It is a malignant tumor of melanocytes, cells which arise from the neural crest and it is considered the fourth most common cancer in individuals between the ages of 0 and 44 years [4]. It can occur *de novo* or from a preexisting lesion usually located in skin areas exposed to the sunlight. The annual incidence has increased dramatically over the past few decades. According to the Italian Cancer Society Report 2006, there were an yearly average of 12.5 new skin Melanoma diagnoses per 100,000 males and 13.1 per 100,000 females, with incidence rates for skin Melanoma remarkably varying across Italy with a decreasing trend moving from North to South (2:1 ratio; total estimated incidence: 6000 new cases per year) [4].

These finding are almost comparable to our results (11.5–12.0 per 100,000 inhabitants) both overall (more than 5.800 cases in 2008) and for Northern/Central/Southern Italian regions. The differences in prevention campaigns and proper/early diagnosis cannot completely explain the existing gap between Northern and Southern Italian regions. At a European level the highest European Standardized Rates (ESR) incidence rates were reported for Denmark in 2010 (21.5 per 100,000 men and 26.1 per 100,000 females), with the lowest incidence observed in Portugal (4.6 per 100,000 males and 6.2 per 100,000 females) [13]. It is important to point out that usually the incidence of Melanoma is higher in women than in men [14]. A recent case control study, including 5700 cases of malignant Melanoma showed a complex relationship between risk of developing this skin tumor and the individual patterns of sun exposure (recreational/occupational), body sites and sunburns [15].

The issue of the latitude where the affected person is living has also been addressed in a recent paper [16]. Thus, environmental and native population-related factors (*i.e.*, skin pigmentation) seem to play a crucial role in the incidence of malignant Melanoma. The prognosis and the treatment of this tumor depends on the type of Melanoma, the patient’s age, the presence or absence of ulceration, the depth of invasion and the nodal status at diagnosis [17]. A prospective study showed the possibility of using ERβ expression as a prognostic indicator of Melanoma; in fact it is evidenced that thin Melanomas show significantly higher ER mRNA levels than thicker lesions [18]. While the majority of patients present with a primary cutaneous malignant Melanoma and are cured by surgical resection alone, metastasis to regional lymph nodes or distant sites occurs in a proportion and is associated with poor long-term survival [19]. Furthermore, in those with visceral metastatic disease, Melanoma is usually rapidly fatal, with an average survival of less than one year, and it is associated with much morbidity [19].

Prognosis from Melanoma is determined by traditional anatomical staging; the risk of relapse from a primary Melanoma correlates with features such as tumor thickness, ulceration, and mitotic rate, and in advanced Melanoma, worsened clinical outcomes are observed in those patients with visceral metastases and those with an elevated lactate dehydrogenase level, presumed to reflect a higher burden of metastatic disease [20]. Similarly, performance status was also found to be a prognostic variable in patients with stage IV Melanoma treated in clinical trials [20]. Palliative systemic therapy is the basis of management for metastatic Melanoma, and until very recently, a global standard was dacarbazine, an alkylating chemotherapy agent. However, metastatic Melanoma is regarded as being insensitive to cytotoxic chemotherapy, as evidenced by response rates to dacarbazine in the order of 10% and no proven survival benefit [21,22,23,24]. Immunotherapy, including cytokine and vaccine treatments, provides the only alternative to chemotherapy but does not benefit the majority of patients, although durable responses have been observed in a small proportion of patients treated with high-dose interleukin-2 [25]. The generally held view that metastatic Melanoma is refractory to systemic treatments was dramatically altered in 2010, when positive Phase III clinical trial results were reported for two novel agents [26]. Both the anti-cytotoxic T lymphocyte-associated antigen-4 antibody, ipilimumab, and the small molecule inhibitor of BRAF, vemurafenib (formerly referred to as PLX4032 and RG7204), were shown to improve overall survival in patients with advanced Melanoma in randomized controlled trials [27].

## 4. Conclusions 

Hospitalizations due to malignant Melanoma in Italy show a decreasing incidence rate from northern to southern Italian Regions, being possibly influenced by environmental and population-related factors. 
